# Circuit life span in critically ill children on continuous renal replacement treatment: a prospective observational evaluation study

**DOI:** 10.1186/cc6965

**Published:** 2008-07-25

**Authors:** Jimena del Castillo, Jesús López-Herce, Elena Cidoncha, Javier Urbano, Santiago Mencía, Maria J Santiago, Jose M Bellón

**Affiliations:** 1 Pediatric Intensive Care Unit. Hospital General Universitario Gregorio Marañón, Universidad Complutense de Madrid, Madrid, Spain; 2 Preventive and Quality Control Service, Hospital General Universitario Gregorio Marañón, Universidad Complutense de Madrid, Madrid, Spain

## Abstract

**Introduction:**

One of the greatest problems with continuous renal replacement therapy (CRRT) is early coagulation of the filters. Few studies have monitored circuit function prospectively. The purpose of this study was to determine the variables associated with circuit life in critically ill children with CRRT.

**Methods:**

A prospective observational study was performed in 122 children treated with CRRT in a pediatric intensive care unit from 1996 to 2006. Patient and filter characteristics were analyzed to determine their influence on circuit life. Data were collected on 540 filters in 122 patients and an analysis was performed of the 365 filters (67.6%) that were changed due to circuit coagulation.

**Results:**

The median circuit life was 31 hours (range 1 to 293 hours). A univariate and multivariate logistic regression study was performed to assess the influence of each one of the factors on circuit life span. No significant differences in filter life were found according to age, weight, diagnoses, pump, site of venous access, blood flow rate, ultrafiltration rate, inotropic drug support, or patient outcome. The mean circuit life span was longer when the heparin dose was greater than 20 U/kg per hour (39 versus 29.1 hours; *P *= 0.008), with hemodiafiltration compared with hemofiltration (34 versus 22.7 hours; *P *= 0.001), with filters with surface areas of 0.4 to 0.9 m^2 ^(38.2 versus 26.1 hours; *P *= 0.01), and with a catheter size of 6.5 French or greater (33.0 versus 25.0 hours; *P *= 0.04). In the multivariate analysis, hemodiafiltration, heparin dose of greater than 20 U/kg per hour, filter surface area of 0.4 m^2 ^or greater, and initial creatinine of less than 2 mg/dL were associated with a filter life of more than 24 and 48 hours. Total effluent rate of greater than 35 mL/kg per hour was associated only with a filter life of more than 24 hours.

**Conclusion:**

Circuit life span in CRRT in children is short but may be increased by the use of hemodiafiltration, higher heparin doses, and filters with a high surface area.

## Introduction

Continuous renal replacement therapy (CRRT) is currently the treatment of choice in critically ill adults and children with acute renal failure, fluid overload, or multiorgan dysfunction as it allows a steady removal of fluid, creatinine, urea, and other substances and produces with less hemodynamic instability than occurs with hemodialysis [1-5]. One of the greatest problems with CRRT is early coagulation of the filters, leading to blood loss, decreased efficacy of the technique, increased costs, and a greater risk of hemodynamic instability in the connection [6,7]. Contact between the blood and an artificial surface is the factor underlying the initiation of coagulation, although other factors such as the number of blood flow reductions [8], hemoconcentration, a high platelet count, turbulent blood flow, and blood-air contact in the air-detection chambers are also involved [9,10]. Few studies have monitored circuit function prospectively in patients on CRRT [9,10]. We conducted a prospective observational study to determine those variables associated with circuit life in critically ill children treated by CRRT.

## Materials and methods

A prospective observational study was performed in a pediatric intensive care unit from 1996 through 2006. The study was approved by the local institutional review board. Due to the characteristics of the study, informed consent of patients was not considered to be necessary. Patients requiring CRRT were included prospectively. Data from those filters that had to be changed because of circuit coagulation were analyzed. The machines used for continuous venovenous renal replacement therapy were the BSM32 (Hospal, Lyon, France) during the first 5 years of the study and then the PRISMA (Hospal). Venous access was secured by inserting a double-lumen catheter into one of the central veins. The size of the catheters depended on the size of the patient. Either polyacrylonitrile AN69 (Hospal) or polysulfone (L.IN.C Medical Systems Ltd, Leicester, UK) hollow-fiber hemofilters were used, depending on the body surface area of the patient and on the pump employed. Commercially prepared, bicarbonate-buffered hemofiltration replacement fluid (Clearflex^®^; Bieffe Medital, Senegue, Spain) was infused prefilter to compensate for fluid losses, which were assessed clinically. Decisions on the use of hemodiafiltration were the responsibility of the treating physician and were based on the patient's need for solute clearance. Age, gender, weight, and diagnoses were recorded on admission to the pediatric intensive care unit and pediatric risk of mortality (PRISM), pediatric index of mortality (PIM), and pediatric logistic organ dysfunction (PELOD) scores, lactic acid, and need for inotropic drug support were recorded at the beginning of the CRRT. The variables analyzed in order to determine their influence on circuit life were catheter and venous access used, blood flow rates, heparin dose, filter surface area, filtration fraction, ultrafiltration rate, and total effluent flow rate (ultrafiltration plus dialysis). The reasons why the filters stopped functioning were collected prospectively; filters removed electively were excluded from the analysis. Anticoagulation was performed according to our protocol. Circuits were primed with 1 L of 0.9% NaCl to which 5,000 IU of heparin was added. Because of active bleeding or severe coagulopathy, anticoagulation was not performed in 15 filters. The rest of the patients were anticoagulated. At the time CRRT was initiated, a heparin bolus of 20 to 50 IU/kg was administered, depending on the baseline activated clotting time (ACT). Patients with a normal baseline ACT received a bolus of 50 IU/kg and this was reduced to 20 IU/kg for a baseline ACT of greater than 200 seconds. This was followed by a continuous heparin infusion via the pre-blood pump port, aiming to maintain an ACT of between 150 and 200 seconds. No other anticoagulation drug was administered.

### Statistical analysis

The statistical analysis was performed using the SPSS (version 15) statistical program (SPSS Inc., Chicago, IL, USA). Analysis of normality was performed with the Kolmogorov-Smirnov test. The chi-square test, Fisher exact test, Mann-Whitney test, Kruskal-Wallis test, and analysis of variance were used to compare the qualitative and quantitative variables and to assess the influence of each factor on circuit life span. A multivariate logistic regression model was performed. Results are expressed as the odds ratio and corresponding 95% confidence intervals. Significance was taken as a *P *value of less than 0.05.

## Results

During the study period, 122 patients were treated with CRRT. The clinical and demographic data of these patients are shown in Table 1. Data were collected on 540 filters, and the analysis was performed using the 365 (67.6%) filters that were changed because of signs of circuit coagulation (the filters were clotted, or there was an important increase in the filter pressure in the CRRT monitor, which suggest coagulation of the filter). The median circuit life was 31 hours (range 1 to 293 hours). The analysis of the influence of each factor studied is presented in Table 2. Filter life was slightly longer in children older than 12 months and with a weight of greater than 10 kg, although this difference did not reach statistical significance. Hemofilter life did not correlate significantly with patient diagnoses, initial creatinine and urea, site of venous access, type of pump, blood flow rates, severity of illness score, need for inotropic drug support, or final outcome, nor were significant differences observed on comparing the surface area of the filters or the size of catheter used. However, significant differences in filter life were found on comparing the use of catheters larger or smaller than 6.5 French and the use of filters with surface areas larger or smaller than 0.4 m^2 ^(Table 2). Mean circuit life proved to be significantly longer with hemodiafiltration than with hemofiltration. Filter life was also longer with a total effluent flow rate of greater than 35 mL/kg per hour, ultrafiltration flow rates of less than 25 mL/kg per hour, and filtration fractions of less than 10%, although these differences did not reach statistical significance. The administration of heparin doses of less than 10 U/kg per hour did not lead to any difference in circuit life span but there was a significant increase in life span with doses of greater than 15 U/kg per hour. There were no bleeding events related to heparin dose. In the multivariate logistic regression analysis, hemodiafiltration, heparin dose of greater than 20 U/kg per hour, filter surface area of 0.4 m^2 ^or greater, and an initial creatinine of less than 2 mg/dL were associated with a filter life of more than 24 and 48 hours. Total effluent rate of greater than 35 mL/kg per hour was associated only with a filter life of more than 24 hours (Tables 3 and 4).

**Table 1:** Data of patients and clinical characteristics (n = 122)

	Median	Range
Age, years	1.4	0.16–22
Weight, kg	8	2.5–85

Diagnoses	Number	Percentage

Cardiac surgery	54	44.2
Sepsis	22	18
Nephropaties	15	12.2
Oncologic diseases	10	8.2
Cardiopathies	8	6.5
Gastrointestinal surgery	3	2.4
Hemolytic-uremic syndrome	3	2.4
Metabolic diseases	3	2.4
Others	4	3.2

CRRT indication		

Acute renal failure	51	42
Fluid overload	22	18
Acute renal failure plus fluid overload	39	32
Tumor lysis	5	4
Others	5	4

	Median	Range

Initial creatinine, mg/dL	2.4	0.1–7.8
Creatinine after 24 hours of CRRT, mg/dL	1.5	0.1–3.9
Initial urea, mg/dL	104	6–290
Urea after 24 hours of CRRT, mg/dL	67.2	11–199

	Number	Percentage

Mechanical ventilation	98	84.5
Inotropic drug support	89	73

	Mean	Standard deviation

PRISM score at the beginning of CRRT	14	8.3
PELOD score at the beginning of CRRT	14.6	8.0
Number of failed organs	3	72% >2 organs

	Number	Percentage

Survival	84	68.9

CRRT, continuous renal replacement therapy; PELOD, pediatric logistic organ dysfunction; PRISM, pediatric risk of mortality.

**Table 2:** Factors associated with the circuit life span

	Circuit life span, hours		*P *value
	
	Mean	Standard deviation	
Age			
<12 months	29.1	32.8	0.33
>12 months	33.3	35.5	
Weight			
<10 kg	28.8	31.9	0.24
>10 kg	34.3	37.0	
Period of time			
1996–2001	27.0	25.0	0.43
2002–2006	32.8	37.4	
Pump type			
PRISMA	31.8	36.1	0.98
BSM32	29.0	27.1	
Catheter size			
Two 4 French catheters	12.5	8.6	
Double-lumen 5 French Double-lumen 6.5 French	25.7 34.2	25.4 37.8	0.07
Double-lumen 8–9 French	22.8	20.8	
Double-lumen 10–11 French	36.1	38.6	
4–5 French	25.0	25.0	0.04
6.5–11 French	33.0	36.3	
Venous access			
Subclavian	34.2	27.6	
Femoral	30.5	34.4	
Jugular	21.2	21.9	0.12
Other	38.2	40.5	
Blood flow rate			
<50 mL per hour	29.9	34.5	0.33
>50 mL per hour	32.8	33.2	
Filter surface area			
0.04 m^2^	27.7	31.2	
0.15 m^2^	20.6	14.5	0.18
0.4 m^2^	43.6	51	
0.6 m^2^	37.7	36.8	
0.9 m^2^	38.2	53.5	
0.04–0.15 m^2^	26.1	28.7	0.01
0.4–0.6–0.9 m^2^	38.2	40.3	
Filter membrane			
Polysulphone	28.5	28.5	0.18
Acrylonitrile	32.0	35.3	
Anticoagulation			
No heparine	31	16.6	0.15
Heparine	31	34.6	
Heparine dose			
<10 U/kg per hour	28.4	28.4	0.08
>10 U/kg per hour	34.5	34.4	
<15 U/kg per hour	28.9	31.5	0.02
>15 U/kg per hour	35.5	38.9	
<20 U/kg per hour	29.1	31.5	0.008
>20 U/kg per hour	39.0	42.7	
Hemodiafiltration			
Yes	34.0	36.6	0.001
No	22.7	24.0	
Ultrafiltration rates			
<25 mL/kg per hour	32.2	35.4	0.10
>25 mL/kg per hour	21.0	17.2	
Filtration fraction			
<10%	31.5	34.6	0.12
>10%	18.3	13.3	
Total effluent flow rate			
>35 mL/kg per hour	34.6	39.6	0.06
<35 mL/kg per hour	25.8	22.9	
Initial creatinine			
<2 mg/dL	32.4 26.5	36.0 22.3	0.39
>2 mg/dL			
Initial urea			
<60 mg/dL	30.9	34.1	0.99
>60 mg/dL	30.4	32.0	
Mortality			
Yes	32.6	36.8	0.94
No	30.2	32.6	
PRISM score			
<15	29.0	29.5	0.95
>14	36.1	41.7	
Lactic acid			
<4 mmol/L	28.7	29.9	0.91
>4 mmol/L	34.7	39	

PRISM, pediatric risk of mortality.

**Table 3:** Multivariate analysis of circuit life span more than 24 hours

Factor	*P *value	Odds ratio	95% CI
Weight <10 kg	0.147	1.914	0.796–4.606
Catheter 6.5–11 French	0.051	1.933	0.996–3.749
Filter 0.4–0.9 m^2^	0.006	3.311	1.407–7.795
Initial creatinine <2 mg/dL	0.009	2.534	1.263–5.088
Heparine dose >20 U/kg per hour	0.031	2.026	1.065–3.852
Hemodiafiltration	0.001	4.162	1.781–9.726
Ultrafiltration <25 mL/kg per hour	0.224	1.811	0.696–4.710
Total effluent flow rate >35 mL/kg per hour	0.029	2.143	1.082–4.245

CI, confidence interval.

**Table 4:** Multivariate analysis of circuit life span more than 48 hours

Factor	*P *value	Odds ratio	95% CI
Weight <10 kg	0.074	2.486	0.916–6.742
Catheter 6.5–11 French	0.093	2.128	0.002–5.134
Filter 0.4–0.9 m^2^	<0.001	6.786	2.396–19.221
Initial creatinine <2 mg/dL	0.011	3.120	1.305–7.460
Heparine dose >20 U/kg per hour	0.007	3.148	1.361–7.279
Hemodiafiltration	0.004	5.473	1.715–17.469
Ultrafiltration <25 mL/kg per hour	0.455	1.812	0.381–8.618
Total effluent flow rate >35 mL/kg per hour	0.109	2.128	0.882–5.134

CI, confidence interval.

## Discussion

The success of CRRT depends in part on maintenance of the extracorporeal circuit [6,11]. CRRT circuit life span is low in adults and children; in our study, we found values similar to those reported previously in children and adults [12-15]. However, there are few studies that have prospectively monitored circuit function in adult patients on CRRT [9,10] and only one studied the influence of vascular access location and size on filter life in children [16]. When life span is evaluated, the main problem appears to be filter coagulation [6,10,11,17-19]. During CRRT, blood passes through an extracorporeal circuit and coagulation is activated. Anticoagulation is therefore necessary. There are a number of agents that can be used to achieve circuit anticoagulation [6,15,17-21] and, of these, heparin continues to be the most widely employed [1,18]. The aim of anticoagulation in our study was to maintain an ACT of between 150 and 200 seconds. We found that the use of higher doses increased circuit life span and we did not observe an increase of bleeding. Other studies have also found that the heparin dose is a significant independent predictor of filter life [10,19]. Although we did not observe any major complications due to heparin use, patients with a risk of bleeding can be managed without anticoagulation [1,22,23]. Some but not all studies have found that the use of sodium citrate increases filter life compared with heparin [6,13,15,17,19,24,25]. In a recent evidence-based review, Oudemans-van Straaten and colleagues [19] recommended that, if bleeding risk is not increased, unfractionated heparin (with an activated partial thromboplastin time of 1 to 1.4 times normal) or low-molecular-weight heparin (anti-Xa 0.25 to 0.35 IU/L) should be used (evidence grade E). CRRT without anticoagulation can be considered when coagulopathy is present (grade D). To our knowledge, no randomized studies in critically ill patients on CRRT have evaluated the effect of catheter site or size. Hackbarth and colleagues [16], in a pediatric registry, found that larger catheter diameter and jugular access are associated with longer circuit life. A short large-caliber catheter is preferable. Smaller-caliber catheters do not permit high flows and are more likely to kink, leading to a greater risk of system malfunction and coagulation. However, a more central position of the tip of the catheter improves flow [6]. In our study, increased filter life was found with larger-caliber catheters, although this factor did not reach statistical significance in the multivariate analysis, nor were differences in filter life found on comparing the different sites of catheter placement, although the simultaneous influence of a number of factors makes it difficult to analyze these results adequately.

Filter life was slightly longer in children over 1 year of age and in those with a body weight of greater than 10 kg, probably due to the larger caliber of the catheters used and the greater surface area of filters; however, the differences were not significant. We found a significant correlation between circuit life span and filter surface area. Although the filter must be chosen according to the patient's age and weight, a larger surface area is associated with a longer life span. This might be explained by the fact that membrane saturation could be delayed by the presence of a larger filter surface area. These filters permit higher blood flows, thus increasing capillary shear forces and reducing protein layering, with the consequent decrease in membrane clotting and the prolongation of filter life [6]. In a prospective study, filters with longer hollow fibers had a longer life and a lower transmembrane pressure than filters with a larger cross-section. This may have been due to a lower blood flow leading to an increase in blood viscosity in a filter with a larger cross-section [26]. Although the type of membrane can also affect filter coagulation, no studies, to our knowledge, have analyzed the effect of this variable during CRRT in critically ill patients. In our study, there were no differences between polysulfone and acrylonitrile membranes, but the surface areas of the filters were different. We found no difference in filter life over the study period or on comparing the two types of machine used. Although more modern machines permit greater control of the process, more accurate adjustment, and have more sensitive alarms, in our experience this has not led to longer filter life. This lack of a difference between the machines could be due in part to the fact that a higher percentage of filters with a smaller surface area were used with the more modern machines. In adults, circuit life has been found to be longer with continuous venovenous hemodialysis than with continuous venovenous hemofiltration [27]. In one study in children, hemodiafiltration showed longer circuit life than hemofiltration [16]. In comparison with hemofiltration, hemodiafiltration permits lower blood flow rates to be used and causes less hemoconcentration to achieve the same solute clearance; this could explain the longer circuit life [6]. In our patients, a total effluent flow rate of greater than 35 mL/kg per hour was associated with a longer filter life, probably due to the use of dialysis. This suggests that continuous venovenous hemodiafiltration can achieve sufficient fluid and substance interchange efficacy with a lower risk of coagulation. However, a prospective survey in children did not find a correlation between circuit survival and CRRT mode [13]. Using lower ultrafiltration rates and lower filtration fractions could prolong circuit life, minimizing the procoagulant effects of hemoconcentration [6]. Although filtration fractions of up to 25% have been used by some groups, we currently use low filtration fractions, though still achieving adequate clearances and azotemic controls. In our study, there were no statistically significant differences in relation to filtration fraction. Another option for reducing the filtration fraction is to administer the replacement fluid before the filter to reduce the hemoconcentration. In two studies, predilution replacement was associated with longer circuit survival [10,28]. In our study, we consistently used predilution replacement and therefore are unable to analyze the effect of this factor. Apart from the dose of heparin, the multivariate study also revealed that a low creatinine at the time of starting CRRT was associated with a longer filter life. This could be due to the fact that patients with a higher creatinine required higher rates of ultrafiltration, which could have led to increased filter coagulation. However, as the comparison was performed using the creatinine at the time of starting CRRT and not with the creatinine level at the time of each change of filter, these results must be viewed with caution.

Our study has certain limitations. It is an 11-year, descriptive, epidemiological study that does not test any intervention and it is possible that some differences could be due to the changes in treatment over time. However, it is prospective in design and aims to increase our understanding of the nature and magnitude of a phenomenon that has been poorly explained up to now. Second, when comparing heparin doses, we did not study the patients' anticoagulation parameters. Although it seems reasonable to consider that correct anticoagulation rather than the dose of heparin could prolong the circuit life span, this conclusion cannot be drawn from our data. In addition, it has not been possible to control the effect of problems with the vascular access on filter life in this study; this is one of the main causes of CRRT system malfunction [8], particularly in infants. Another factor that could not be studied is staff training. This is a determinant factor in the early recognition of and response to pump alarms, having a significant effect on filter life [6].

## Conclusion

We conclude that, in children on CRRT, filter life can be increased by the use of hemodiafiltration, high heparin doses, and filters with a large surface area.

## Key messages

• Circuit life span in continuous renal replacement therapy in children is short.

• Filter life can be increased by the use of hemodiafiltration, high heparin doses, and filters with a large surface area.

## Abbreviations

ACT = activated clotting time; CRRT = continuous renal replacement therapy.

## Competing interests

The authors declare that they have no competing interests.

## Authors' contributions

JdC and JL-H conceived the study and participated in the study design, data collection and analysis, and drafting of the manuscript. EC, JU, SM, and MJS participated in the data collection and analysis and drafting of the manuscript. JMB participated in the design of the study and performed the statistical analysis. All authors read and approved the final manuscript.
